# A protocol for longitudinal monitoring of individual building occupants and their environments

**DOI:** 10.1371/journal.pone.0274015

**Published:** 2022-09-23

**Authors:** Graham Coulby, Adrian K. Clear, Oliver Jones, Suzanne McDonald, Alan Godfrey

**Affiliations:** 1 Faculty of Engineering and Environment, Department of Computer and Information Sciences, Northumbria University, Newcastle Upon Tyne, United Kingdom; 2 School of Computer Science, College of Science and Engineering, National University of Ireland Galway, Galway, Ireland; 3 Ryder Architecture, Newcastle Upon Tyne, United Kingdom; 4 Centre for Clinical Research, The University of Queensland, Brisbane, QLD, Australia; 5 General Practice Clinical Unit, The University of Queensland, Brisbane, QLD, Australia; Yeungnam University, REPUBLIC OF KOREA

## Abstract

Buildings account for approximately 40% of the energy consumption across the European Union, so there is a requirement to strive for better energy performance to reduce the global impact of urbanised societies. However, energy performant buildings can negatively impact building occupants (e.g., comfort, health and/or wellbeing) due to a trade-off between airtightness and air circulation. Thus, there is a need to monitor Indoor Environmental Quality (IEQ) to inform how it impacts occupants and hence redefine value within building performance metrics. An individualised study design would enable researchers to gain new insights into the effects of environmental changes on individuals for more targeted e.g., health interventions or nuanced and improved building design(s). This paper presents a protocol to conduct longitudinal monitoring of an individual and their immediate environment. Additionally, a novel approach to environmental perception gathering is proposed that will monitor environmental factors at an individual level to investigate subjective survey data pertaining to the participant’s perceptions of IEQ (e.g., perceived air quality, thermal conditions, light, and noise). This protocol has the potential to expose time-differential phenomena between environmental changes and an individual’s behavioural and physiological responses. This could be used to support building performance monitoring by providing an interventional assessment of building performance renovations. In the future it could also provide building scientists with a scalable approach for environmental monitoring that focuses specifically on individual health and wellbeing.

## 1. Introduction

Strategies for carbon neutrality, such as Net Zero Carbon, are promoting a focus on the built environment to challenge current best practices, making buildings (and cities) more sustainable. Congested city commuting and transportation emissions in major cities are often the focus of attention, but buildings are by far the largest energy consumer within the European Union, accounting for 40% of the total energy consumption [1]. This is unsurprising given the increased amount of time urbanised societies spend indoors. A recent focus on energy consumption is changing how the construction industry manages buildings across lifecycles, but the narrow focus on energy performance is leading to buildings that negatively impact the health and wellbeing of building occupants [2]. Reductions in energy consumption are often achieved by sealing buildings to prevent air leakage (drafts), which reduces air circulation and causes contaminants to accumulate, posing a risk to occupant health [3].

There is a need for increased energy performance within buildings to protect the health and wellbeing of future generations, but energy performance modifications have been found to reduce environmental quality [4–6] so this needs to be balanced with the health and wellbeing of current building occupants. The global climate crisis has often dominated the narrative, but the SARS-COV-2 pandemic has increased the focus on monitoring and controlling indoor environments to better regulate Indoor Environmental Quality (IEQ) e.g., increased ventilation [7]. However, comprehensive IEQ monitoring requires the measurement of a wide range of outcomes, comprising of subjective comfort factors *(e*.*g*., *noise*, *privacy*, *or control over the environment)* and objective environmental conditions *(e*.*g*., *the thermal environment or indoor air quality)* [8].

Measurement of comfort is a complex field of study that largely relies on self-reporting mechanisms for capturing how occupants experience environmental conditions [9]. These data can be highly subjective and influenced by factors such as personal health conditions and clothing. Yet, standardised approaches to comfort measurement (ISO 7730 [10], BS EN 16798 [11] and ASHRAE 55 [12]) generalise the views and experiences of occupant populations as opposed to measuring at an individual level. The disconnect between environmental measurements and occupant comfort can lead to low accuracy when predicting the comfort of individuals [13].

Study methods can also impact the symmetry between comfort factors and environmental conditions. For example, Andargie *et al*. [8] note that studies were found to either measure IEQ at a different time to when surveys were conducted or that objective and subjective outcomes were measured in isolation with assumptions being made about the unmeasured outcomes. Thus, there is a need for research that conducts measurement of environmental conditions and comfort factors simultaneously, while measuring IEQ at an individual level. However, research-grade measurement devices lack the scalability required to conduct such research and advocating such technologies on projects *(at scale)* can be challenging [14]. Alternatively, low-cost monitoring has been identified as a requirement for future research where the technology could be used to develop scalable solutions that can monitor buildings at a more granular level, providing individualised monitoring [2].

The Internet of Things (IoT) is a disruptive technology that is increasing the feasibility of low-cost monitoring solutions in research by increasing the accessibility of sensor technologies and reducing development costs [15]. Accessible low-cost sensors enable the development of multimodal monitoring solutions that can be tailored to the specific needs of a project [16]. Consequently, IoT technologies can be regarded as a development toolkit for researchers to create more engaging and reactive research [15]. Since these devices can be made at a low-cost with multimodality, they could be deployed at scale and left in situ for continuous synchronous monitoring of IEQ and comfort. However, comfort changes over time and is influenced by external factors such as seasons/weather/outdoor environmental changes so comfort must be modelled to adapt to these changes [13].

Longitudinal monitoring may have the potential to expose causal relationships between measured variables and time-differential behavioural phenomena [17,18]. Longitudinal tracking of seasonal data (e.g., weather and air pollution) obtained from local weather stations could augment IEQ data to enable adaptive modelling approaches. For example, an adaptive personal comfort model involves predicting an individual’s comfort instead of evaluating the averaged responses of a population, but it also involves evaluating the impact time and/or external factors have on comfort [13]. Longitudinal observational studies that focus on an individual as the unit of analysis can be useful for identifying causal relationships between measured variables [19] and can identify time-differential phenomena that relate to those causal links [18]. Due to the longitudinal and observational nature of those studies, it is possible to observe naturally occurring phenomena over time [18]. A previous review of literature [2] identified mature approaches for monitoring objective environmental outcomes but found many studies utilised complex and expensive research-grade measurement devices. This often results in short study periods where devices are placed at a single location *(in multi-occupant spaces)*, resulting in coarse-grained spatial data. However, IEQ can vary in buildings, floors, and rooms in a non-uniform manner. Therefore, the distance between an individual building occupant and a monitoring device can lead to inconsistencies between the conditions measured and the conditions experienced by the individual. Furthermore, approaches used to capture occupant experiences often generalise the views of multiple occupants that are experiencing different parts of the same space and potentially different IEQ conditions [13]. The review in [2] also identified a gap in knowledge and a need for a paradigm shift in IEQ research, whereby individual participants become the unit of analysis in building performance studies. This presents a need for scalable low-cost measurement technologies and data capture approaches that are suited to individualised assessments. Accordingly, this protocol aims to present a methodological approach to monitoring individuals in an IEQ context to complement a body of work [15,20–22] surrounding the identification, development, testing and validation of scalable low-cost technologies that could address the technological challenges of this knowledge gap.

This protocol presents methods, technologies and workflows that will be used to monitor an individual’s environmental conditions within the spaces they occupy as well as objective measurements of an individual’s immediate IEQ. Consequently, the unit of analysis will not be the building, but rather individual responses to changes within a building. Thus, multiple environmental settings could be monitored to understand how occupants respond to both environmental changes and changes in environmental conditions. It is hypothesised that, by collecting data from multiple sources, the methodologies outlined in this protocol could help to gain new insights into the impact of environmental changes on individuals.

## 2. Methods

### 2.1 Study design

In this study, a mixed methods approach will be adopted to explore the interactions and relationships between an individual’s physiological and behavioural responses to environmental changes. Additionally, a comparative analysis of (traditional) survey data and sensor data will be adopted to measure the relationship between perceptions of environmental conditions and objective environmental measurements (from passive environmental sensors). That approach will enable outcomes to be measured at an individual level and not require a large sample size or generalised findings of a population.

**Fig 1:**
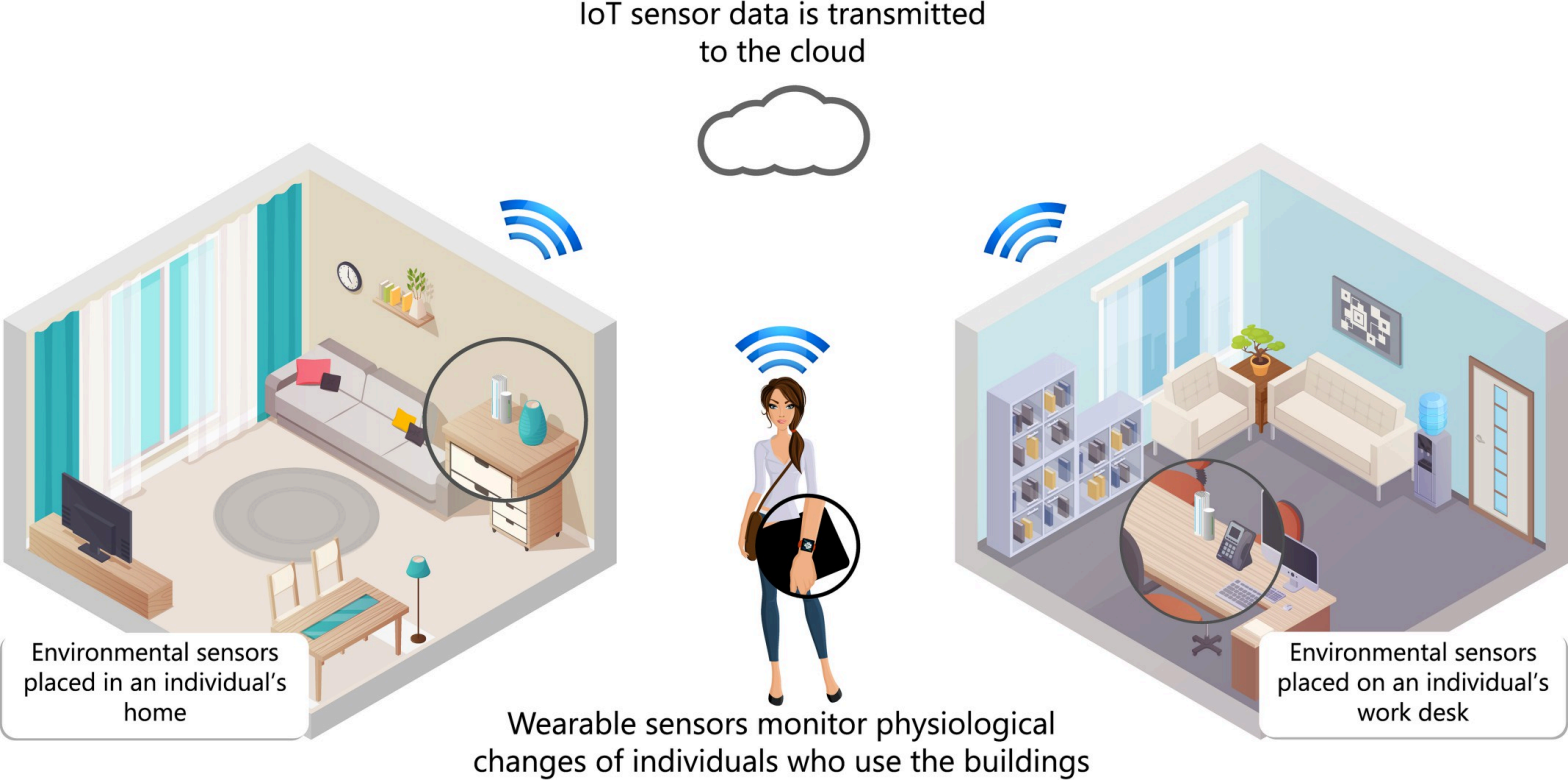
Diagram of the study setup and passive sensor configuration.

The protocol described here will quantify regular physical activity levels (including walking) and heart rate alongside environmental factors with the aim to identify causal relationships between sedentary and active behaviour during environmental changes *(Fig 1*). Given the relationships between objective measurements and perceived IEQ, it is hypothesised that the individualised approach could also be used to identify relationships between perceptions and measurements. Therefore, it is possible that these methods may also be applied as a novel approach for conducting adaptive personal comfort models in future research. This has the potential to provide a holistic methodology for personalised building performance assessments.

### 2.2 Study setting

This protocol is designed to simultaneously monitor different environments with passive environmental sensors and focus on the individual to pose a unique opportunity for IEQ research. This could involve monitoring multiple spaces within a single building that an individual would occupy *(e*.*g*., *occupant’s office*, *meeting rooms or breakout areas)*, or it could involve monitoring multiple buildings that an individual would occupy *(e*.*g*., *home and office)*. This protocol could be applied to either scenario. To mitigate the risks SARS-COV-2 could have on the study, this protocol will be applied to monitoring multiple environments within a home setting. Consequently, a participant is required that works from home with a home office that has distinct separation from their living area.

### 2.3 Eligibility criteria

There are no inclusion/exclusion criteria pertaining to participant health for this study, but the participant will be of working age and will predominantly work from their office. The participant should be willing to wear a fitness tracker, which will monitor physical activity levels and heart rate during the working day *(09*:*00–17*:*00)* throughout the study period. The participant should also be willing to have sensors installed in their home and office, which will passively monitor IEQ and transmit data to the cloud *(Fig 1)*.

### 2.4 Interventions

Since this protocol outlines a longitudinal observational study, there will be no interventions required.

### 2.5 Sample size

This is an observational study that will focus on drawing conclusions about an individual over time with the aim of exposing causal relationships between measurement outcomes. Therefore, only one participant is required for this study, but that participant will be monitored longitudinally to ensure statistical power to detect relationships between variables comes from repeated measurement of those variables over time.

### 2.6 Participant timeline

This protocol outlines the design of a 16-week longitudinal observational study that will use the individual as the unit of analysis. The exact timeline for the participant will also include three additional days that will be used for an initial meeting with the participant as well as one day on either side of the study period for mobilising and demobilising the study.

### 2.7 Initial meeting

The participant will receive full details of the study and provide written informed consent prior to the study commencing. The participant will be given the opportunity to discuss and negotiate the pragmatics of the study procedures, within the boundaries of an approved ethics application. This will help to reduce the burden on the participant and ensure the study does not interfere with day-to-day activities.

The participant will receive an Apple Watch and two identical sets of environmental sensors. One set of sensors will be placed near the participant’s office workstation and the other within the living room of their home. Since the study will monitor the participant’s home, sensors will be connected on a dedicated 4G mobile network, to ensure no security vulnerabilities are exposed to their personal network.

## 3. Outcomes

### 3.1 Primary outcomes

The primary outcome for this study will be **the physiological and behavioural responses to environmental changes.** The **study will** assess the variance in heart rate, walking and activity levels (Table 1) using exponential moving averages which will be calculated from minute-by-minute measurements and used to determine if there is a change in the timeseries.

**Table 1:** Dependant variables of the primary outcomes.

Outcome	Source	Sample Period
Walking Data (*e*.*g*., Steps/Distance/Speed)	Apple Watch	After event
Heart Rate	Apple Watch	After event
Activity Levels (*e*.*g*., calories)	Apple Watch	After event

^‡^ AppleWatch does not record at a fixed sample rate but instead records aggregated data retrospectively after event bouts.

### 3.2 Predictors of the primary outcome

This protocol will also explore a series of secondary outcomes (Table 2). These outcomes will provide objective measurements for each of the environmental outcomes, which will be obtained from validated and passive, multimodal, environmental sensors [16]. The secondary outcomes will also explore physiological and behavioural changes, which will be obtained from the Apple Watch.

**Table 2:** Covariates to predict the primary outcomes.

Outcome	Source	Sample Period
Temperature	Passive IEQ Sensor	1 minute
Humidity	Passive IEQ Sensor	1 minute
Air Pressure	Passive IEQ Sensor	1 minute
Light	Passive IEQ Sensor	1 minute
Noise	Passive IEQ Sensor	1 minute
Carbon Dioxide (CO_2_)	Passive IEQ Sensor	1 minute
Particulate Matter up to 2.5*μm* in diameter (PM_2.5_)	Passive IEQ Sensor	1 minute
Local Weather	OpenWeatherMap API ^†^	1 hour
Outdoor Air Pollution	OpenWeatherMap API^†^	1 hour

^†^ Historical data will be captured retrospectively from the OpenWeatherMap API, the API supports real-time connections and is therefore scalable, but this is not required for this study.

## 4. Data collection, management, and analysis

Quantitative environmental data will be obtained from passive sensors in-situ and physiological data will be captured from a wearable wrist-worn fitness tracker. To adhere to recommendations on study length, data will be collected for a total of 16 weeks to ensure it is sufficiently longitudinal for individualised timeseries analysis [19]. Data on perceptions of environmental conditions will be captured using short, real-time surveys. Sensor data from this study will be used to investigate the data captured from surveys during analysis.

### 4.1 Data collection methods

#### 4.1.1 Measuring physiological and behavioural responses

An Apple Watch Series 3 will be used to capture quantitative physiological outcomes, which will be stored on the participant’s phone during the study via the iOS Health app. These data will also be combined with passive environmental sensor data to monitor heart rate and activity levels in relation to environmental changes. The wearable will be used to examine if physiological changes correlate with environmental changes and to assess whether environmental changes impact activity levels. The wearable will also monitor physiological responses to comparable environmental quality captured from different environments.

#### 4.1.2 Measuring IEQ changes

IEQ sensors will need to be placed near the study participant, ensuring the participant’s perceptions of IEQ are reflective of the data being captured. For this study, it is important to ensure any identified solutions can be pragmatically deployed outside of research. Therefore, low-cost IoT sensors (identified as fit-for-purpose in a previous study [16]) will be used to measure IEQ in both environments.

IoT sensors should be considered a toolkit to develop bespoke multimodal monitoring equipment that is tailored to researcher requirements. As a result, the specific sensors used to measure the IEQ outcomes (**Table 2**) will likely be project specific. For example, monitoring measured/perceived noise levels would not be required if the participant were deaf. The same would apply to light levels for blind participants. Therefore, appraising and identifying sensors *(or discussing how they should be configured)* is deemed outside the scope of this protocol. However, a previous study was conducted [16] that outlined this process and presented a bespoke multimodal device that was developed using low-cost sensors and IoT technologies. This multimodal device will be used to capture all the outcomes listed in Table 2 to provide a quantitative measurement of indoor environmental quality within the home and home office. Though, researchers should note that the IoT market is rapidly developing [15], so they should conduct a review of the market prior to selecting sensors for their study. Thus, the specific sensors have not been detailed here, as the study outcomes are deemed more important than the specific sensors used to capture the data. Please see [16] for details of the sensors deployed in this study.

#### 4.1.3 Measuring IEQ perceptions

Perceived environmental quality and comfort assessment will be captured using surveys informed by traditional pen and paper-based approaches. However, surveys can often be burdensome, generally asking occupants to rate perceptions against scales, such as e.g., temperature from “Cold” to “Hot” [12]. Ratings are typically based on subjective responses that will likely differ between occupants, meaning data could only be used for generalising the perceptions of a population. Qualitative scale-based responses may also lead to situations where occupants are unable to accurately determine the difference between e.g., “Slightly Warm” and “Warm”, meaning test-retest reliability could be affected. By complimenting survey data with sensor data, it could be possible to achieve greater meaning from comfort assessments, whereby sensor data could be used to reinforce data pertaining to perceived IEQ [23]. If the environmental conditions are known to the researcher (from passive sensors), the mechanism for capturing perceptions may be altered to reduce the number of subjective responses required by participants. This could reduce survey burden and allow for more prolonged/longitudinal feedback to be captured.

Here, an alternative solution is proposed that exploits the reinforcement of multimodal IEQ outcomes. Instead of asking occupants to rate the environmental factors using a scale, occupants will instead be asked if they are *e*.*g*., *“too cold”*, *“too hot”* or *“comfortable”*. In isolation, these data are highly subjective and less meaningful than traditional scales. Yet, the combination of these data with quantitative data from environmental sensors mean that this approach could help identify an individual’s perception of *e*.*g*., hot and cold, without burdening the occupant with multi-page surveys.

Surveys will investigate the participant’s perceptions of IEQ. To ensure data from surveys can be combined with passive sensor data, questions will be related to the specific measurements being captured by IEQ sensors. Since this study is intended to last many months, the survey is designed to be short and quick to complete, so regular feedback can be captured without study fatigue. Since high frequency survey capture can be burdensome to the participant, the exact time and frequency of data capture should be negotiated during the initial meeting with the participant.

Table **3** presents a list of questions and responses that will be used to determine the perceived environmental quality outcomes.

**Table 3:** Automated survey questions and responses.

Outcome (Perceived)	Question	Response 1	Response 2	Response 3
Temperature	How is the temperature?	Too Cold	Comfortable	Too hot
Humidity	How is the humidity?	Too Dry	Comfortable	Too humid
Light	How is the light?	Too Dark	Comfortable	Too light
Noise	How is the noise?	Too Quiet	Comfortable	Too noisy
Air Quality	How is the air circulation?	Too Draughty	Comfortable	Too stuffy
Air Quality	Is it Dusty?	Yes	No	-
Air Quality	Are the any odours?	Yes	No	-

### 4.2 Data management

#### 4.2.1 AppleWatch

**Fig 2:**
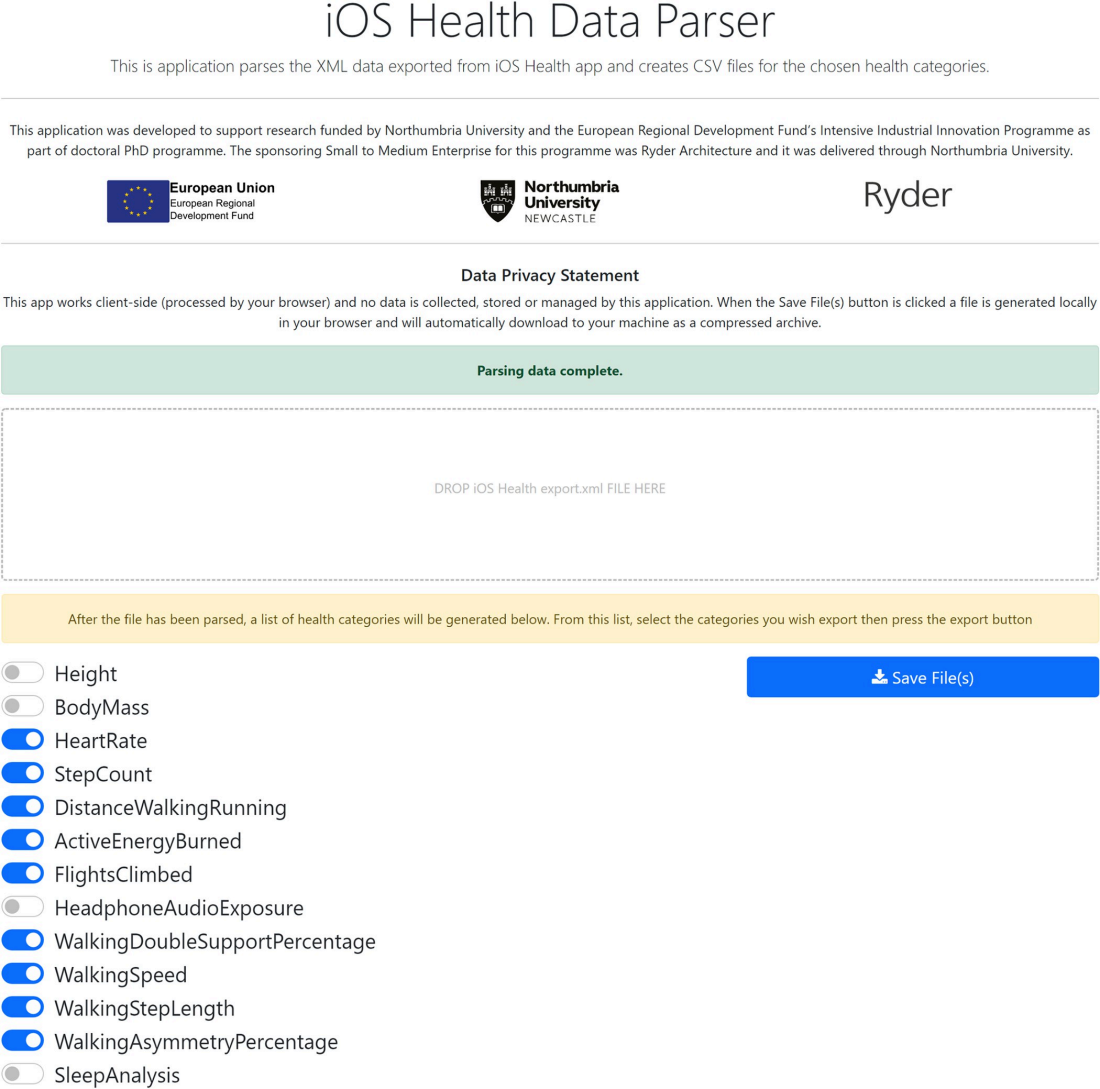
Screenshot of iOS Health Data Parser application.

At the end of the study, the participant will export their data using the Apple Health app and will be provided access to a web app [24] that has been developed for this study. The app was developed as the iOS health app that exports all data and includes personal data that would be unrequired for this study. Therefore, the app will provide the participant with a transparent system for converting data into the format required here (CSV). The app has been designed so that all processing is done on the participant’s computer to ensure data are not uploaded to the internet. The app allows participants to choose which data to export, allowing them to remain in control over what they are sharing (Fig 2). Oftentimes, these data can still be used to identify the device owner as Apple device names are typically named after the account holder e.g., Graham’s iPhone/AppleWatch. Consequently, device names in the exported files will be simplified to iPhone/AppleWatch before the data is stored in Microsoft OneDrive.

#### 4.2.2 Multimodal IEQ sensors

For this study, data from multimodal IEQ sensors will be transmitted to the cloud using ThingSpeak^™^, an open-source, cloud-based, IoT platform developed around MATLAB^®^. To ensure rate limits are not reached, data will be transmitted and stored on the platform every 40 seconds. Previous work [15] identified ThingSpeak^™^ as fit-for-purpose in IEQ monitoring. The platform supports Secure HyperText Transfer Protocol (HTTPS) and Message Queuing Telemetry Transport (MQTT) transmission protocols. MQTT will be selected for this study as it is one of the most popular transmission protocols used in consumer IoT devices [25]. It is also most used on enterprise-level cloud platforms such as Microsoft Azure, Amazon Web Services and Google Cloud platform, as a result of the bandwidth differences [15].

#### 4.2.3 Comfort assessment surveys

Depending on the participant preferences, survey data will either be captured using paper-based forms that will be collected at the end of the study or will be captured digitally throughout the study. Either way, the data will be stored digitally in a Google sheets spreadsheet and will be exported to Microsoft OneDrive upon study completion.

### 4.3 Statistical methods

Traditional statistical techniques are not applicable for the analysis of longitudinal data collected from an individual because they assume data are independent. Autocorrelation *(where observations from one measurement may be wholly/partially influenced by another measurement)* may be present in timeseries data, which can lead to underestimation or overestimation of standard errors [17].

To conduct the analysis, moving averages will first be calculated to determine changes in the dependent and independent variables. A ten-point exponential moving average will track the variables in Tables 1 and 2 to form a new dataset (*Table 4*) with a degree of smoothing that is exponentially waited to the most recent variables. This will highlight significant movements across the timeseries and enable changes in variables to be analysed. The new dataset will contain both the original data and the moving average for each variable, so that the original data can be analysed at points where significant changes are highlighted.

**Table 4:** Variables to be included in dynamic regression model.

Dependent Variables	Independent Variables
Heart Rate (bpm) Steps (n) Energy Burned (kCal)	CO_2_ (ppm) eCO_2_ (ppm) Temperature (°C) Humidity (%) Light (lux) Noise (dBA) PM_2.5_ (μ/m^3^)

^†^ All variables will be analysed as exponential moving averages of the data captured at the frequencies outlined in Tables 1 and 2.

**Fig 3:**
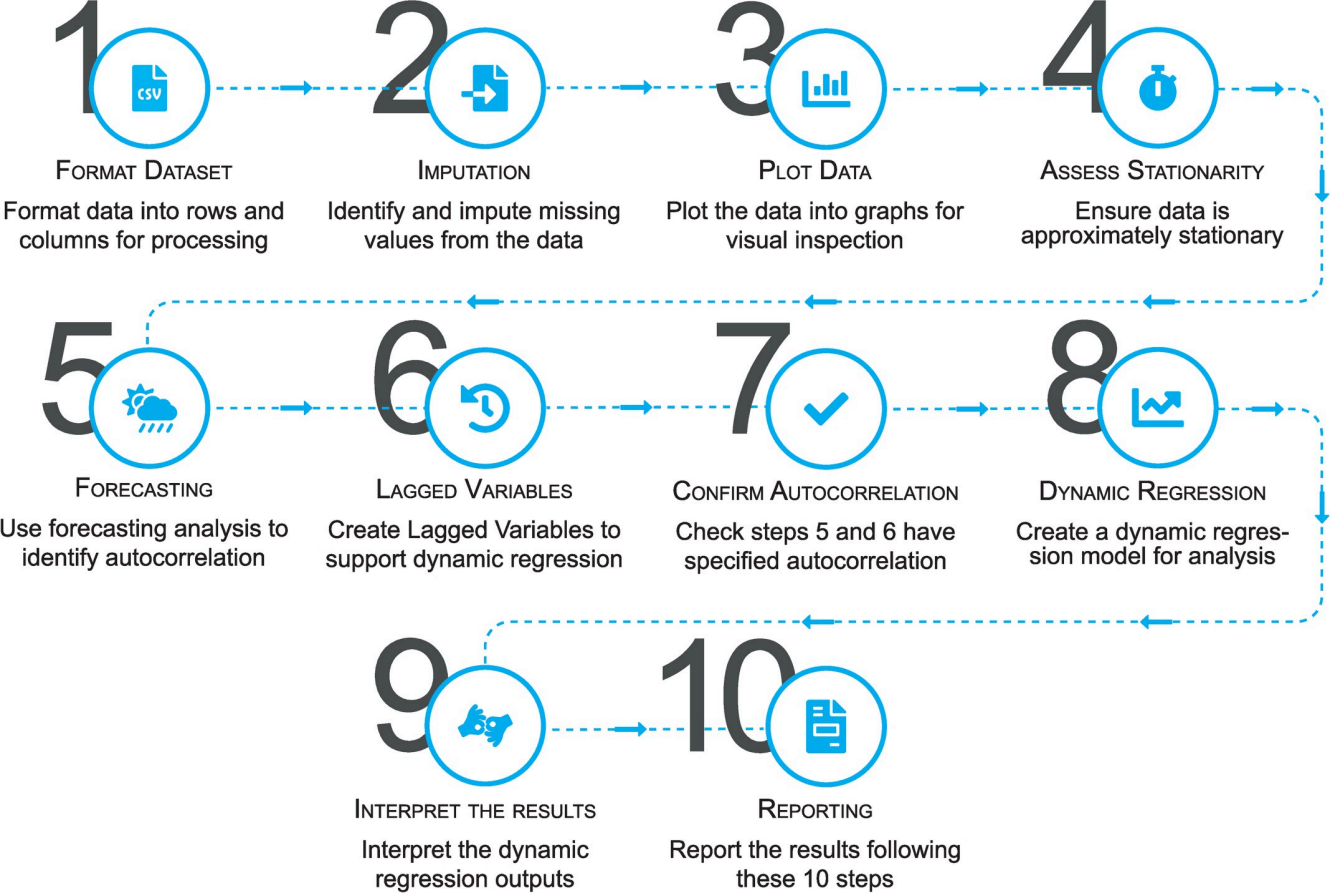
Flowchart representation of the methodological approach within this study for analysing timeseries data with dynamic regression.

Dynamic regression analysis will be conducted following a 10 step process (*Fig 3*), to identify causal relationships between the dependant variables (*Table 1*) and the independent covariates (*Table 2*). This process will involve preparing the dataset ready for dynamic regression modelling, which will include formatting the dataset, assessing how stationary the data is and evaluating the extent of autocorrelation in across the data, which are important first steps in dynamic modelling [26]. Fig 3 provides an indicative high-level overview of the steps that will be involved for the purposes of this study when conducting the statistical analysis, but readers are directed to a comprehensive tutorial for conducting such analyses in IBM SPSS Statistics for a more in-depth outline of the statistical methods involved [18].

## 5 Ethics & dissemination

### 5.1 Research ethics approval

The protocol outlined in this paper was granted ethical approval from the Northumbria University Research Ethics Committee *(Submission Reference*: *20481)*.

### 5.2 Informed consent

Informed consent will be obtained from the participant by the principal investigator during the initial meeting. The participant will consent to the researchers to obtain data from wearable sensors, environmental sensors and surveys. The participant will consent to the terms of the study as negotiated during the initial meeting.

### 5.3 Confidentiality

Confidentiality will be maintained throughout this study. While it will not be possible to blind the participant from the research or vice versa, all data collected by the researchers will be anonymous or anonymised prior to analysis. Demographic data on the participant will be included in the write-up and analysis of data, but this be presented in a way that provides no means to identify a natural living person.

## 6 Discussion

The protocol outlined here has the potential to explore a unique avenue of IEQ research. Monitoring IEQ in an individual context presents opportunities to not only gather spatially dense IEQ data within buildings, but the data captured will be representative of what is experienced by individuals near the monitoring equipment. By using emergent low-cost technologies, researchers can develop scalable and more objective/insightful and personalised monitoring solutions that could be used to address a range of challenges for building scientists [16]. It is hypothesised that the protocol presented here could provide unique contributions to building science research by addressing current gaps in literature around the effect environmental changes within buildings have on individual building occupants.

## 7 Contribution

The proposed approach of using personal assessment by longitudinally measuring an individual, could be used to examine relationships between environmental conditions, activity behaviour and physiological changes *(e*.*g*., *change in heart rate)*. This may help identify individual thresholds of comfort in future work. For example, if these thresholds are known with a degree of confidence, it could be possible to remove the need for survey feedback entirely *(or heavily reduce the data capture frequency)*. Furthermore, longitudinal capture of these thresholds could also be used to inform personal comfort models trained with machine-learning, comfort thresholds with real-time data capture [13].

If environmental measurements detect changes in IEQ outside of an individual’s comfort thresholds, it is possible that those data could drive building management systems that manage environmental conditions at a local level [27]. These data could potentially enable the development of automated systems that can provide personalised actionable feedback to occupants based on environmental conditions. Systems exist that provide actionable advice to building occupants based on building information and IoT-based environmental monitoring, but there is a need for a wider context to understand the more subjective factors of IEQ [28]. With additional context, such systems could provide steps to control the IEQ, if conditions rise or fall outside of an occupant’s comfort threshold and if they have control over the environment. If occupants do not have control over the environmental conditions *(e*.*g*., *in a shared office environment)*, the system could provide *e*.*g*., alternative work locations, based on measurements obtained from other sensors.

It should be noted that when applying this protocol to a single case study, the perceptions of the participant could not be used to evaluate building performance as they would be too subjective in isolation. To conduct such a study, multiple, synchronous, individualised studies would need to be conducted on a wider building population. It is still suitable to use this individualised approach for group studies, but each individual would be measured as a single case [18]. The results could still be used to get indications of average comfort levels, but the individual focus in the data would provide added value compared to traditional group studies. Where traditional group studies would generalise the views/opinions of the population, multiple individualised studies have the potential to expose variations among the study sample. However, while multiple individualised studies could be used to assess generalised findings within a population, each single-case study should be treated as its own study. In doing so, causal relationships could be assessed between intra-case variables, but cross-examination should not be conducted on inter-case variables. Instead, the results from each case could be used to form a new dataset, which could be evaluated as a population study. This is particularly important with regard to the use of wrist-worn fitness trackers, as the heart rate sensors can report variations when used to measure different demographics [29]. These variations would not impact individual single-case studies but would likely impact wider population comparisons if measured within an inter-case, multivariate analysis. It is hypothesised that the protocol outlined here could provide a holistic methodology for understanding personalised thresholds of comfort while gaining quantitative insights into the effect buildings and environments have on occupants. This has the potential to provide a deeper understanding of individual building occupants and move away from the generalised measurements of occupant populations seen within current building standards [10–12]. This approach could also help to acknowledge variations across individuals, which could add value to building performance assessments.

## 8 Limitations of study

The small sample size in single-case studies could be perceived as a limitation and is often faced with barriers and resistance in practice [30]. However, single-case studies are specifically designed with a view to using longitudinal timeframes in the examination of an individual, which can provide greater insights on changes in health and behaviour over time, when compared with studies of larger sample sizes [19]. Alternatively, multiple individualised studies can run simultaneously with results used to evaluate how generalised the findings of individual cases are [17]. Upon validating the approach and evaluating the findings of this protocol, future research could scale the methods up to multiple individualised studies.

The use of in-situ sensors outlined in this protocol means that this protocol is ideally suited to office workers, who would generally work from a fixed location. Researchers wishing to apply this protocol to workers with a more mobile profession would need to develop multimodal measurement devices that are portable and potentially wearable. This was deemed outside the scope of this protocol as it presents many complications including, *(but not limited to)* how sensors are calibrated to deal with participants navigating between indoor and outdoor environments. The participant selection process will account for this limitation, but it should be considered in future research. Where participants have more active jobs, which involve moving between spaces or changing working locations, more in-situ sensors could be used to account for these transitions.

## 9 Conclusion

From longitudinal monitoring of individuals, time-differential outcomes can be observed that in future work could help to determine individualised thresholds of comfort. Additionally, robust methods for data collection and analysis of individuals could expose causal relationships between environmental changes and physiological responses. These comfort levels could be used to train machine learning models and aid the development of automated feedback mechanisms that provide individualised actionable advice to building occupants. The approach proposed here has the potential to counter generalisation in occupant comfort studies by exposing variations in research groups while providing a deep understanding of the effect environmental changes have on building occupants at an individual level. This could have practical implications for building owners as it could provide them with a better understanding of how buildings could be adapted to suit individual variations in comfort. Overcoming generalisations in comfort assessment could extend this protocol to work as an interventional model for evaluating the impact energy performant buildings have on individuals. For example, longitudinally monitoring individuals during future energy performance renovation projects. In this way, this protocol could have the potential to identify causal pathways between energy performant buildings and the health and wellbeing of building occupants.
